# Supplementary material for: Exposure to air pollutants contributes to increased rate of neovascular age-related macular degeneration in Israel
Source: PLoS One. 2025 Apr 18;20(4):e0317436. doi: 10.1371/journal.pone.0317436 (PMC12007707; doi:10.1371/journal.pone.0317436)
Supplement: Table S1 — For initial air pollution data (N = 1,214 localities). (DOCX) [file pone.0317436.s001.docx]

Statistic Mean St. Dev. Min Pctl(25) Pctl(75) Max

CM NO2 ugm3 8.434 5.194 0.800 4.434 11.775 29.413

CM NOx ugm3 10.553 6.603 1.684 5.564 14.248 41.170

CM CO ugm3 142.78 25.233 114.512 126.827 150.562 291.703

CM O3 ugm3 76.685 7.815 55.200 70.799 82.312 102.779

CM PM10 ugm3 38.272 2.296 32.173 36.477 39.873 46.210

CM PM25 ugm3 17.897 1.625 13.200 16.703 19.194 22.238

CM SO2 ugm3 0.664 0.974 0.100 0.100 0.940 8.439
