# Supplementary figures and images for: Exposure to air pollutants contributes to increased rate of neovascular age-related macular degeneration in Israel
Source: PLoS One. 2025 Apr 18;20(4):e0317436. doi: 10.1371/journal.pone.0317436 (PMC12007707; doi:10.1371/journal.pone.0317436)

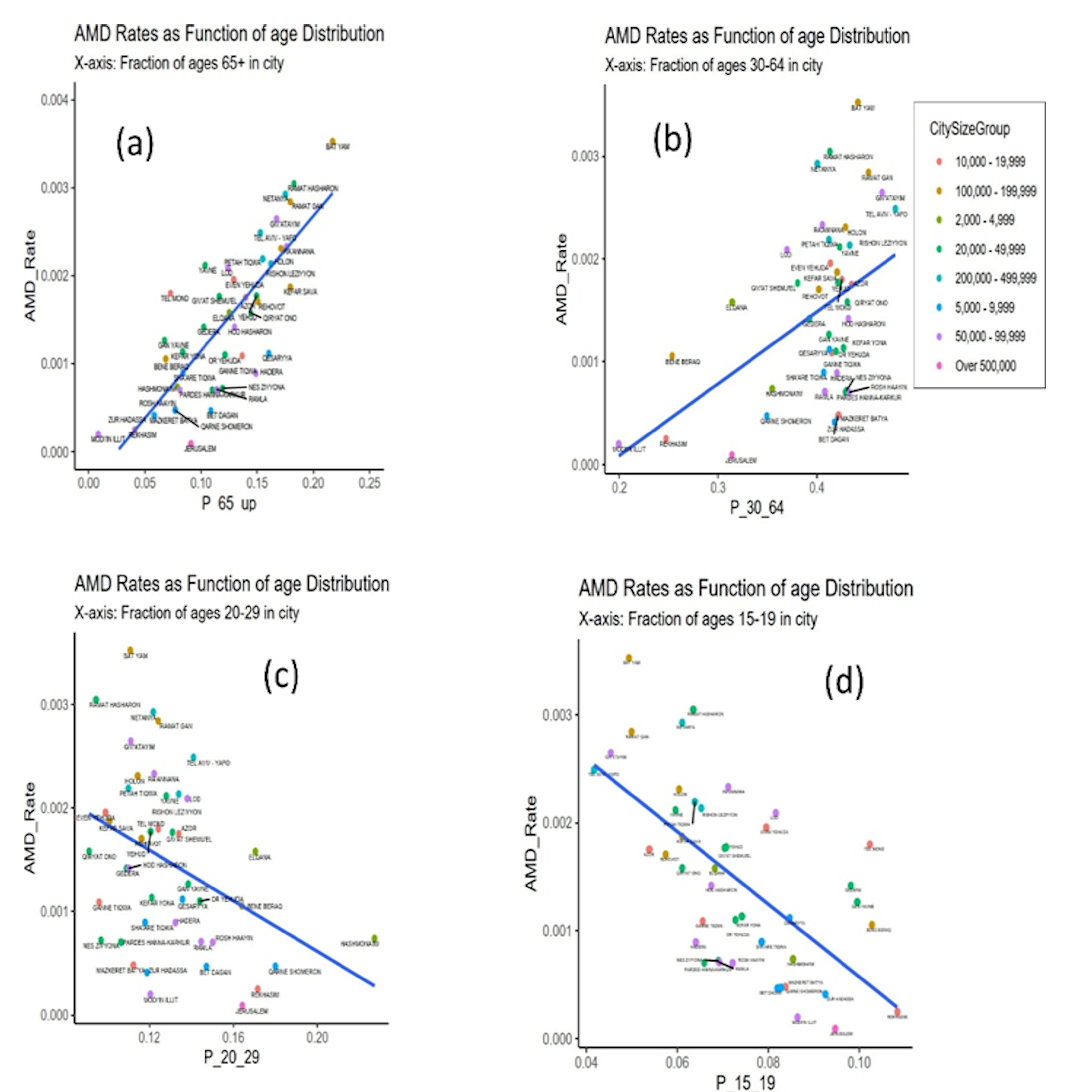

Supplement: Fig S1 — Rates of AMD in different localities across Israel. (PNG) [file pone.0317436.s002.png]

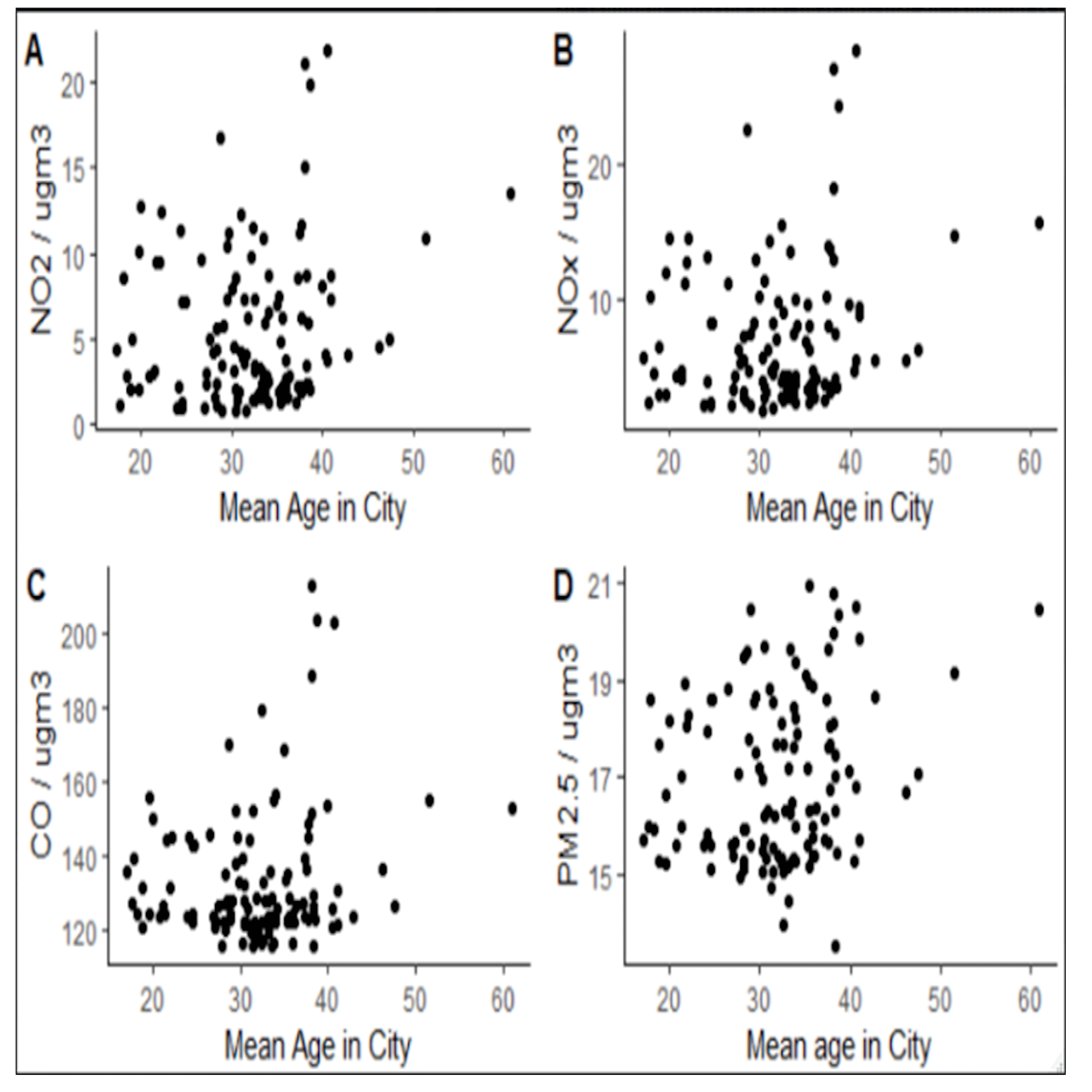

Supplement: Fig S2 — (A) No2, (B), NOx, (C) CO, (D) PM2. Also, t-test were performed to check if cities with older populations have significantly more air pollution. For all seven air pollution particles, we reject this possibility. (PNG) [file pone.0317436.s003.png]

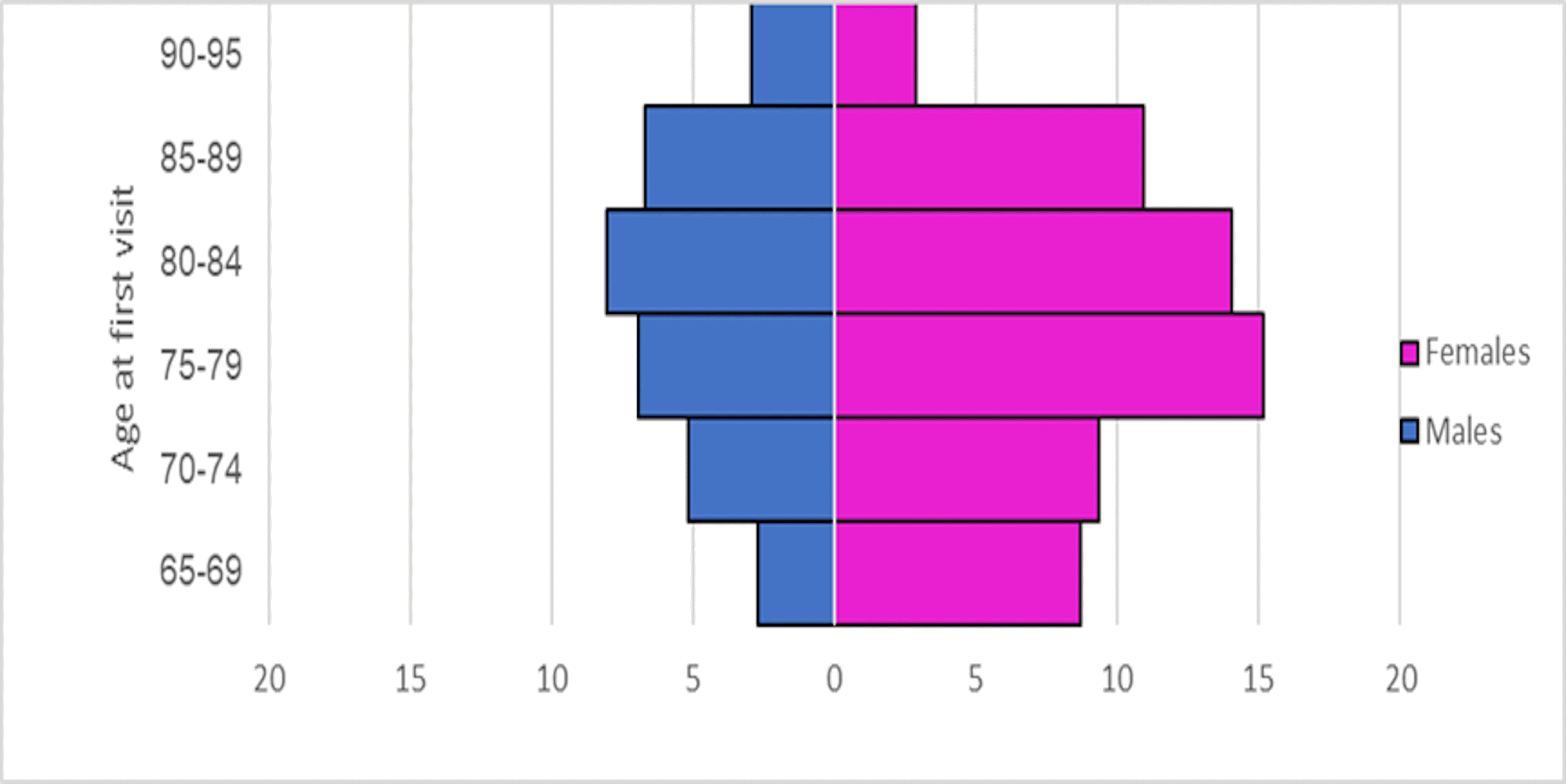

Supplement: Fig S3 — We find gender is rather equally distributed between the cities. Nevertheless, as seen in the image below, more women in our data suffer from AMD in age ranges of 75-90. This could also be due to different life span between genders. (PNG) [file pone.0317436.s004.png]

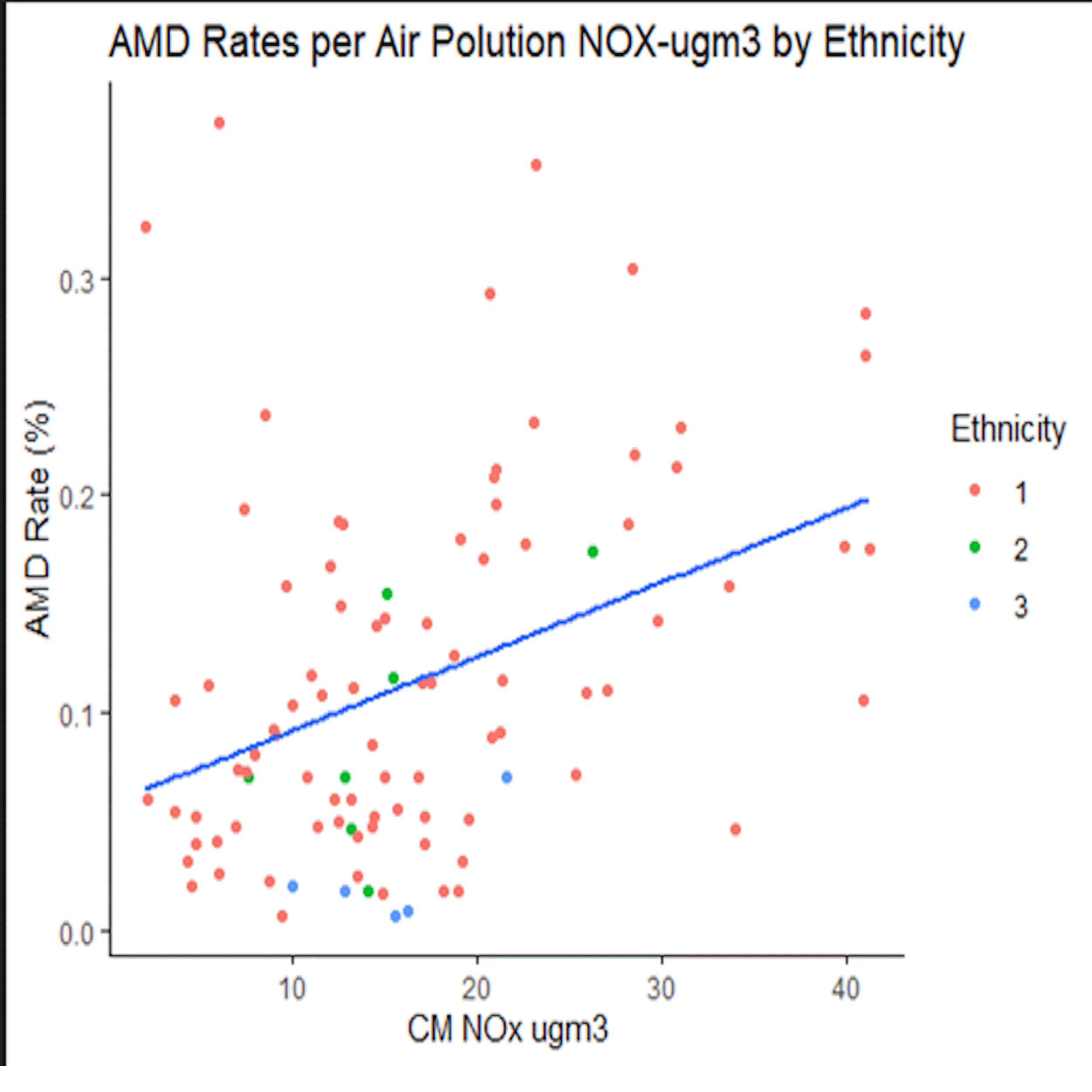

Supplement: Fig S4 — While there are restrictions on ethnicity labelling in medical records in Israel. We do however have language-based clues for ethnicities. (1) represent Jewish towns (where the spoken language is Hebrew). (2) are mixed towns (where ethnicities are mixed, and the spoken language are Arabic and Hebrew, i.e., the population in a mixture of Arabs and Jews, (3) are towns where the spoken language is mostly Arabic, and ethnicities are mostly of Arab origins. One should note that the spoken language is not a clear ethnical recognition, as over 60% of Jewish population is of middle eastern origin. (PNG) [file pone.0317436.s005.png]

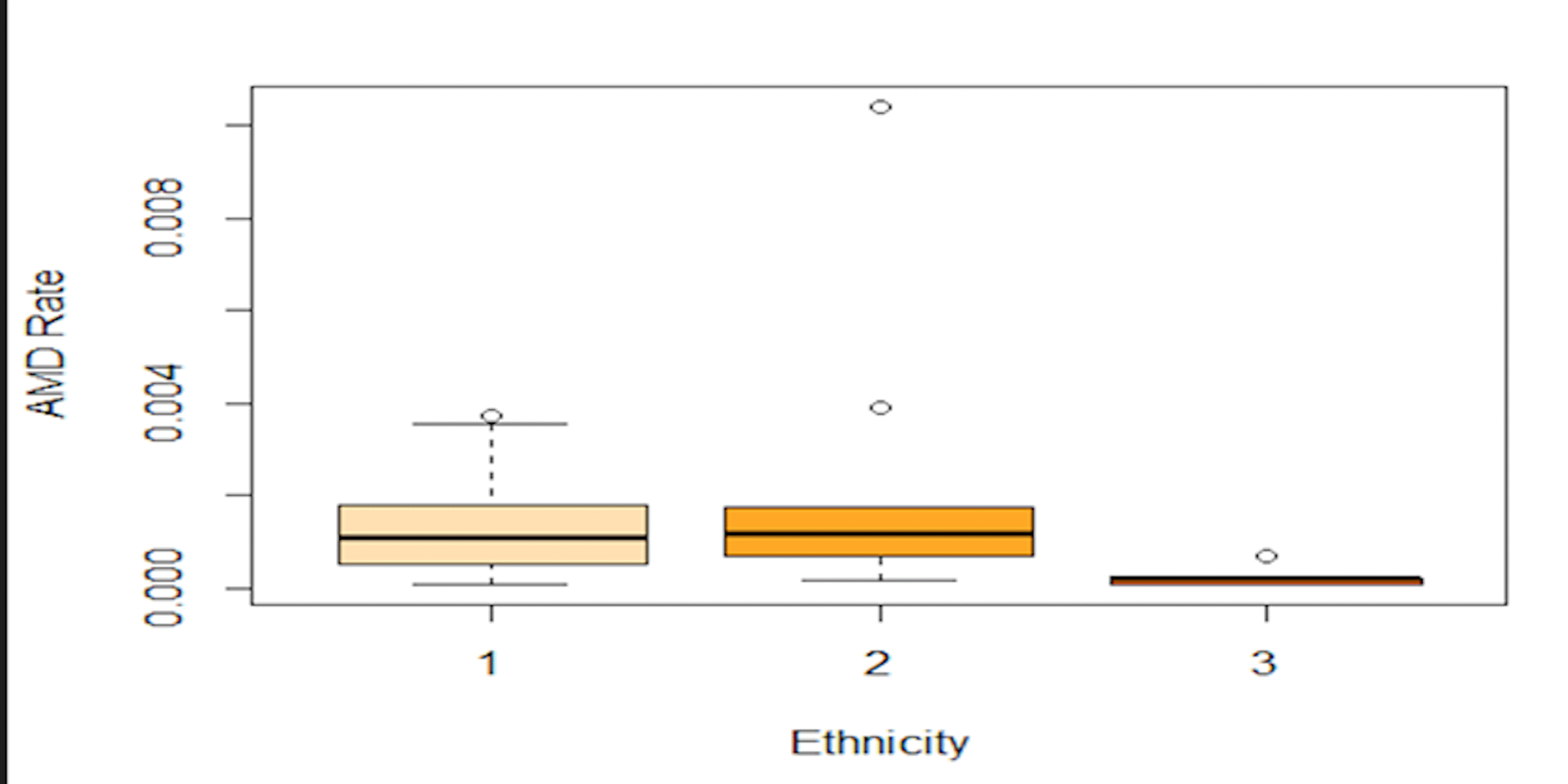

Supplement: Fig S5 — While it seems like AMD is less prevalent in Arab towns, the data set of Maccabi health care is also balanced toward Jewish towns (which are more common in Maccabi health care). Further research is required to conclude this claim on a larger sample. (PNG) [file pone.0317436.s006.png]

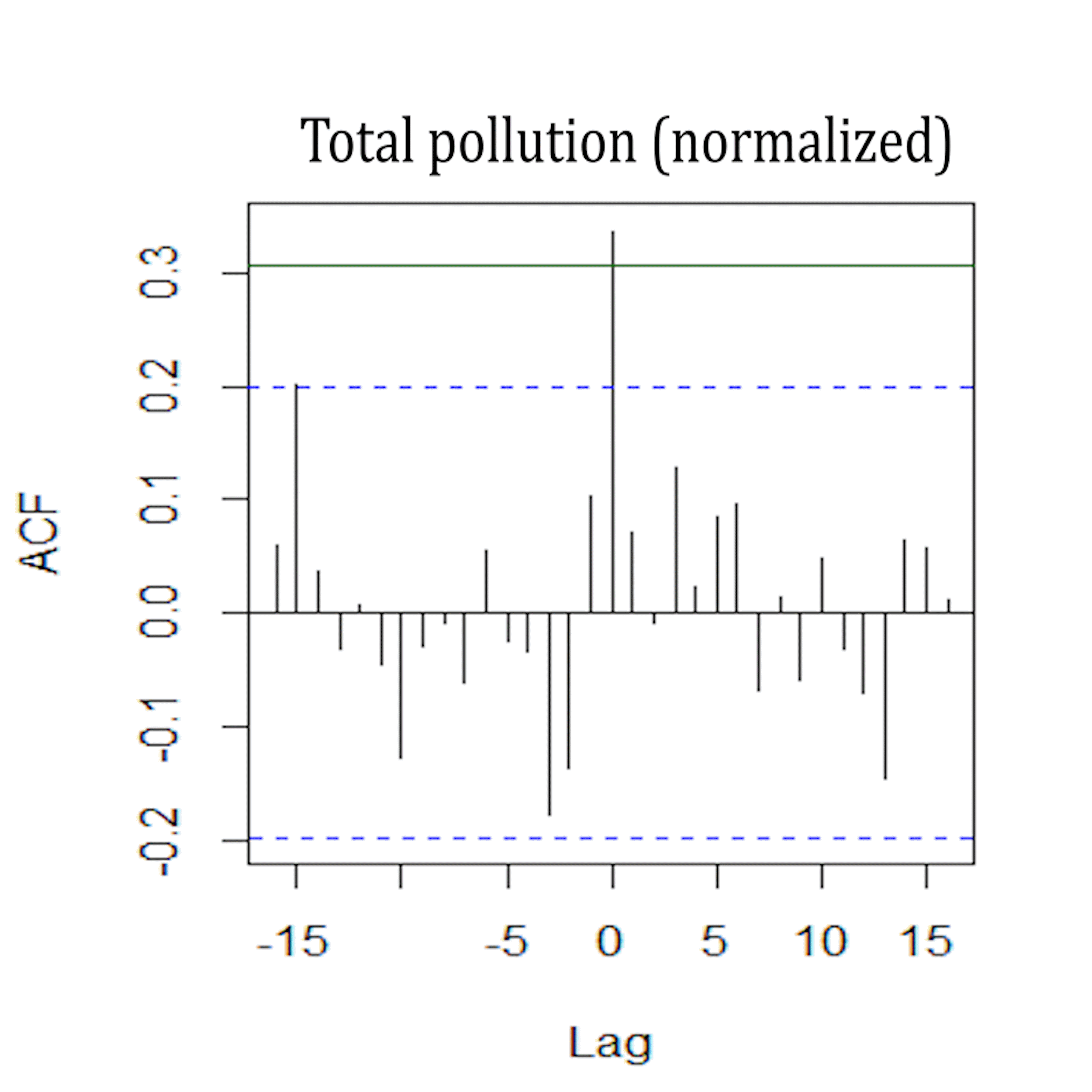

Supplement: Fig S6 — Stronger correlation is clearly observed at LAG = 0, when city air pollution and AMD rates are aligned. There are restrictions on a full ethnicity labelling in medical records in Israel. We do however have language-based clues for ethnicities. (1) represent Jewish towns (where ethnicities can be highly varied). (2) are Arab towns, where ethnicities are mostly middle eastern. (3) are mixed towns, where again, ethnicities are mixed. While it seems as AMD is less prevalent in Arab towns, the data set is balanced toward Jewish towns (which are more common in Maccabi health). Further research is required to conclude this claim on a larger sample. (PNG) [file pone.0317436.s007.png]
