# Supplementary material for: Exposure to air pollutants contributes to increased rate of neovascular age-related macular degeneration in Israel
Source: PLoS One. 2025 Apr 18;20(4):e0317436. doi: 10.1371/journal.pone.0317436 (PMC12007707; doi:10.1371/journal.pone.0317436)
Supplement: Attachment — Submitted filename: plos one_AMD.docx [file pone.0317436.s008.docx]

AMD is a severe problem related to vision loss. There are many factors which might contribute AMD but age is the most important factor. Here in this article the author is trying to relate AMD with air pollutant. Since air pollution is on the rise and the author has shown how these air pollutant has direct relation with AMD, this kind of study is very important. However the author has not mentioned about the age of the patients. It will be important to see if there are any patient with AMD relatively at young age. It is also interesting to see if the author has any data with AMD and air pollutant in relation to the sex of the patient.
