# Supplementary material for: Exposure to air pollutants contributes to increased rate of neovascular age-related macular degeneration in Israel
Source: PLoS One. 2025 Apr 18;20(4):e0317436. doi: 10.1371/journal.pone.0317436 (PMC12007707; doi:10.1371/journal.pone.0317436)
Supplement: Attachment — Submitted filename: Response to reviewers22.05.24.docx [file pone.0317436.s010.docx]

# Response to reviewers

Thank you for the time and effort to read and comment our manuscript - PONE-D-23-40090R1, "***Exposure to air pollutants contributes to an increased rate of neovascular age-related macular degeneration in Israel*".**

Thank you also for your valuable comments, which helped us improve our manuscript.

In our study, we evaluate the relations between several air pollutants the prevalence of Age-related macular degeneration (AMD) in over 90 cities in Israel. We find clear evidence for air pollution influence on the prevalence of AMD. This generative eye disease is expected to become more common as people age and in the future.

Following the reviewer`s comments, we added important additional analysis that improve our claim that air pollution can be associated with growing rates of AMD.

Mainly, after considering the reviewer`s comments, we added an important analysis that inspects if higher AMD rates are simply since older people live in larger cities (that tend to be more polluted). We reject this hypothesis.

We thank the reviewers for this idea, since this analysis suggested by the reviewers strengthen our article.

There was an images where copyrights issues might have arisen. We redraw this image from scratch to overcome possible copywrites issues.

We declare herby that "The funders **had no role in study design, data collection and analysis, decision to publish, or preparation of the manuscript**." .

We thank again the reviewers for the time and effort spent to read and improve our manuscript and look forward to hearing back from you soon.

With kind regards,

Alon Sela (on behalf of the authors).

Journal Requirements:

1. When submitting your revision, we need you to address these additional requirements.

Please ensure that your manuscript meets PLOS ONE's style requirements, including those for file naming. The PLOS ONE style templates can be found at 
<https://journals.plos.org/plosone/s/file?id=wjVg/PLOSOne_formatting_sample_main_body.pdf> and 
<https://journals.plos.org/plosone/s/file?id=ba62/PLOSOne_formatting_sample_title_authors_affiliations.pdf>

2. Thank you for stating the following financial disclosure: 
Ariel-Assuta mutual research grant ARC2. 
 
Please state what role the funders took in the study.  If the funders had no role, please state: "The funders had no role in study design, data collection and analysis, decision to publish, or preparation of the manuscript." 
If this statement is not correct you must amend it as needed. 
Please include this amended Role of Funder statement in your cover letter; we will change the online submission form on your behalf.

We added the sentence to the cover letter."

3. We note that the grant information you provided in the ‘Funding Information’ and ‘Financial Disclosure’ sections do not match.

When you resubmit, please ensure that you provide the correct grant numbers for the awards you received for your study in the ‘Funding Information’ section.

Answer - the grant was a bi-organization research grant ACRC2. The grant was give to the two researchers from both organizations. The corrected method of writing emphasized this aspect.

4. In the online submission form, you indicated that Data are available from the Alon Sela [alonse2012@gmail.com](mailto:alonse2012@gmail.com) upon request.
The medical data is available only on an aggregative level (city level) due to patient care privacy.
The code and the aggregated data is available in a designated repository https://osf.io/zvtae/ which fit PLOS ONE open access requirements.
 
All PLOS journals now require all data underlying the findings described in their manuscript to be freely available to other researchers, either a. In a public repository, b. Within the manuscript itself, or c. Uploaded as supplementary information.
This policy applies to all data except where public deposition would breach compliance with the protocol approved by your research ethics board. If your data cannot be made publicly available for ethical or legal reasons (e.g., public availability would compromise patient privacy), please explain your reasons on resubmission and your exemption request will be escalated for approval.

We uploaded all the code and the data to the Open Science Repository. The code and data are found at <https://osf.io/zvtae/>

Please note that due to the privacy issues, we can only upload the data on an aggregative format, and we uploaded this data as well as air pollution records. The code used to analyze the data is also in the repository.

5. When completing the data availability statement of the submission form, you indicated that you would make your data available on acceptance. We strongly recommend all authors decide on a **data sharing plan before acceptance,** as the process can be lengthy and hold up publication timelines. Please note that, though access restrictions are acceptable now, your entire data will need to be made freely accessible if your manuscript is accepted for publication. This policy applies to all data except where public deposition would breach compliance with the protocol approved by your research ethics board. If you are unable to adhere to our open data policy, please kindly revise your statement to explain your reasoning and we will seek the editor's input on an exemption. Please be assured that, once you have provided your new statement, the assessment of your exemption will not hold up the peer review process.

**Answer** - We made the OSF data repository status as pending awaiting approval. It will be approved upon acceptance or if needed before.

6. Please amend either the title on the online submission form (via Edit Submission) or the title in the manuscript so that they are identical.

Answer – both titles were changed to the title below.

**7. We note that Figure 1 in your submission contain map images which may be copyrighted. All PLOS content is published under the Creative Commons Attribution License (CC BY 4.0), which means that the manuscript, images, and Supporting Information files will be freely available online, and any third party is permitted to access, download, copy, distribute, and use these materials in any way, even commercially, with proper attribution. For these reasons, we cannot publish previously copyrighted maps or satellite images created using proprietary data, such as Google software (Google Maps, Street View, and Earth). For more information, see our copyright guidelines:** [**http://journals.plos.org/plosone/s/licenses-and-copyright**](http://journals.plos.org/plosone/s/licenses-and-copyright)**.
 
We require you to either present written permission from the copyright holder to publish these figures specifically under the CC BY 4.0 license, or remove the figures from your submission:
 
a. You may seek permission from the original copyright holder of Figure 1 to publish the content specifically under the CC BY 4.0 license.**  
 
**We recommend that you contact the original copyright holder with the Content Permission Form (**[**http://journals.plos.org/plosone/s/file?id=7c09/content-permission-form.pdf**](http://journals.plos.org/plosone/s/file?id=7c09/content-permission-form.pdf)**) and the following text:
“I request permission for the open-access journal PLOS ONE to publish XXX under the Creative Commons Attribution License (CCAL) CC BY 4.0 (**[**http://creativecommons.org/licenses/by/4.0/**](http://creativecommons.org/licenses/by/4.0/)**). Please be aware that this license allows unrestricted use and distribution, even commercially, by third parties. Please reply and provide explicit written permission to publish XXX under a CC BY license and complete the attached form.”**
 
**Please upload the completed Content Permission Form or other proof of granted permissions as an "Other" file with your submission.**
 
In the figure caption of the copyrighted figure, please include the following text: “Reprinted from [ref] under a CC BY license, with permission from [name of publisher], original copyright [original copyright year].”

**Answer** - The image was regenerated from scratch based on open maps of the Israeli Government <https://www.govmap.gov.il/?c=204000,595000&z=0&lang=en>

The air pollution labels were manually denoted on this map. The image now does not violate now any copywrite.

 
b. If you are unable to obtain permission from the original copyright holder to publish these figures under the CC BY 4.0 license or if the copyright holder’s requirements are incompatible with the CC BY 4.0 license, please either i) remove the figure or ii) supply a replacement figure that complies with the CC BY 4.0 license. Please check copyright information on all replacement figures and update the figure caption with source information. If applicable, please specify in the figure caption text when a figure is similar but not identical to the original image and is therefore for illustrative purposes only.
The following resources for replacing copyrighted map figures may be helpful:
 
USGS National Map Viewer (public domain): <http://viewer.nationalmap.gov/viewer/>
The Gateway to Astronaut Photography of Earth (public domain): <http://eol.jsc.nasa.gov/sseop/clickmap/>
Maps at the CIA (public domain): <https://www.cia.gov/library/publications/the-world-factbook/index.html> and <https://www.cia.gov/library/publications/cia-maps-publications/index.html>
NASA Earth Observatory (public domain): <http://earthobservatory.nasa.gov/>
Landsat: <http://landsat.visibleearth.nasa.gov/>
USGS EROS (Earth Resources Observatory and Science (EROS) Center) (public domain): [http://eros.usgs.gov/#](http://eros.usgs.gov/)
Natural Earth (public domain): <http://www.naturalearthdata.com/>

[Note: HTML markup is below. Please do not edit.]

Reviewers' comments:

Reviewer's Responses to Questions

**Comments to the Author**

1. Is the manuscript technically sound, and do the data support the conclusions?

The manuscript must describe a technically sound piece of scientific research with data that supports the conclusions. Experiments must have been conducted rigorously, with appropriate controls, replication, and sample sizes. The conclusions must be drawn appropriately based on the data presented.

Reviewer #1: Yes

Reviewer #2: Partly

2. Has the statistical analysis been performed appropriately and rigorously?

Reviewer #1: Yes

Reviewer #2: I Don't Know

**Answer** – all code and data were uploaded to the Open Science data repository [osf.io/6htbk](https://osf.io/6htbk/)

3. Have the authors made all data underlying the findings in their manuscript fully available?

The [PLOS Data policy](http://www.plosone.org/static/policies.action#sharing) requires authors to make all data underlying the findings described in their manuscript fully available without restriction, with rare exception (please refer to the Data Availability Statement in the manuscript PDF file). The data should be provided as part of the manuscript or its supporting information, or deposited to a public repository. For example, in addition to summary statistics, the data points behind means, medians and variance measures should be available. If there are restrictions on publicly sharing data—e.g. participant privacy or use of data from a third party—those must be specified.

Reviewer #1: Yes

Reviewer #2: No

All datasets and R code used to publish this work have been checked and uploaded to the OSF Open Science Foundation data repository: <https://osf.io/zvtae/>

The uploaded data was only on an aggregative form (as is required to reproduce the work). This it to ensure full data privacy compliance.

4. Is the manuscript presented in an intelligible fashion and written in standard English?

PLOS ONE does not copyedit accepted manuscripts, so the language in submitted articles must be clear, correct, and unambiguous. Any typographical or grammatical errors should be corrected at revision, so please note any specific errors here.

Reviewer #1: Yes

Reviewer #2: Yes

5. Review Comments to the Author

Please use the space provided to explain your answers to the questions above. You may also include additional comments for the author, including concerns about dual publication, research ethics, or publication ethics. (Please upload your review as an attachment if it exceeds 20,000 characters)

Reviewer #1: AMD is a severe problem related to vision loss. There are many factors which might contribute AMD but age is the most important factor. Here in this article the author is trying to relate AMD with air pollutant. Since air pollution is on the rise and the author has shown how these air pollutant has direct relation with AMD, this kind of study is very important. However the author has not mentioned about the age of the patients. It will be important to see if there are any patient with AMD relatively at young age. It is also interesting to see if the author has any data with AMD and air pollutant in relation to the sex of the patient.

**Answer** - Thank you for this comment.

For gender, following this important comment, we added this plot of the gender distribution histogram to the SI of the article.

Also after inspecting the gender differences in the different cities, we find gender is rather equally distributed between the cities. Nevertheless, as seen in the image below, larger fraction of women in our data suffering from AMD in age ranges of 75-90.

An important comment of the reviewer was related to ages distributions in cities. Indeed, it could be that the effect we found is only due to the fact that older population prefer to live in large cities, and thus that large cities which are more polluted in general, will have more AMD, not because of the air-pollution but because it has an elder population.

**This issue is indeed a critical issue, since if such case is true, we only show an artifact of old people preference and not a read effect.**

We inspect this topic and reject the hypothesis. We added the following section and am additional image (to the SI) to support our claim. We thank the reviewer for this important comment!

...

"*One possibility that needs to be overruled is that this is simply due to older people preferring larger cities (which are more polluted generally). To check this possibility, we calculated first the mean ages in each city. We then plot these city mean ages  against the major air pollution particles.*

*We could not find any evidence that cities with higher mean population ages are more polluted cities. We also validated this issue statistically. We separated the localities into two groups: One consisting of cities with an above average age and the second with those with below average ages. We then compare (t-test two sided) the mean levels of air pollution particles between the two groups. The p-values of these 7 t-tests are: 1. p-val=0.63 (No2), 2. p-val=0.55 (Nox), 3. p-val=0.29 (CO), p-val=0.90 (O3), p-val=0.68 PM10, p-val=0.38 (PM2.5), p-val=0.12 (So2). As a p-value < 0.05 reflects a significant difference, based on these tests, we reject the hypothesis that cities with older citizens also have higher levels of air pollution. We conclude therefore that air pollution is likely to be the cause of the higher prevalence of AMD, and in any case, this is not due to polluted localities consisting of older habitants. "*

Mean ages at different localities and the air particle concentration. (A) No2, (B) Nox, (C) CO, (D) PM2. Also t-test were performed to check if cities with older populations have significantly higher air pollution. For all seven air pollution particles, we reject this option.

Reviewer #2: I would like to thank you for your efforts on the manuscript entitled “Exposure to air pollutants contributes to an increased rate of neovascular age-related macular degeneration in Israel".

**Answer**: Thank you for the effort to review our work!

Please see my comments below:

MAJOR COMMENTS

**Comment 1**
There is very limited patient information available for this study. There is no information on smoking status nor BMI. Age is the main known risk factor for AMD. Gender is also known but it might be due to women reaching older ages.

**Answer: We added to the SI of the work an image showing the population distribution by gender.** As for race factors, the medical records of Assuta cannot and do not include race due to concern of privacy and inappropriate use. We did find however race related factor which is the type of population in the city. We found a separation of localities into 3 population-based types: (1) localities where language of treatment is purely Hebrew, mixed, and Arabic and Hebrew, and pure Arabic language. We can refer from these to 3 types of localities in terms of population: Mostly Jewish origins, mixed cities, mostly Arab cities.

One should note that in terms of genetic and race, over 60% of Hebrew speaking people are of origin Arab countries, and thus the Hebrew speaking localities does not imply race in the common way.

Also, it is important to note that in the sample of cities that have over 300 Macabi patients and over 20 AMD patients, the only an Arab population is not large enough even if its statistically significant, since only Arab population are less common in Macabi health system.

As to BMI and smoking, the data does not contain this information.

**Figure S4**. AMD rates per ethnicity. There are restrictions on a full ethnicity labelling in medical records in Israel. We do however have language-based clues for ethnicities. (1) represent Jewish towns (where ethnicities can be highly mixed). (2) are mixed towns (Arabs and Jews), where ethnicities are mixed. (3) are Arab towns, where ethnicities are mostly middle eastern.

**Figure S5.** While it seems like AMD is less prevalent in Arab towns, the data set is balanced toward Jewish towns (which are more common in Maccabi health care). Further research is required to conclude this claim on a larger sample.

Comment 2
Lines 99-101 states that, “Records of patients with neovascular AMD who received intravitreal anti-VEGF injections were retrieved from Assuta Medical Centres’ Eye Clinic, where members of Maccabi Healthcare Services are treated”.
The above statement indicates that the patients included in the study were receiving anti-VEGF therapy. Therefore, it is important to include data/information on the treatment status from routine ocular examinations, as that will significantly influence the data. For example, what percentage of these patients were responding to the therapy and showing improvement etc.

**Response:** Thank you for your comment. The purpose of this study was to assess the correlation between air pollution and diagnosis of neovascular AMD . In order to identify these patients, we included patients with the diagnosis of AMD ( ICD 9 XXX) that also received treatment with intravitreal anti VEGF injections.

The current treatment in our institution includes four FDA-approved anti-VEGF drugs– Ranibizumab, Aflibercept, brolucizumab and Faricimab. There is also a much less expensive fifth drug that is FDA-approved for treating cancer - Bevascizumab - which is frequently used to treat nvAMD^[1]^. In many countries, including Israel. It is chosen as a first-line treatment, and a switch to the other more expensive drugs is considered only when it fails. Evaluation of the efficiency of each drug is not an easy analysis mainly since the more expensive drugs are given only where the deterioration prevention is not sufficient.

Comment 3
This study is limited to information on area level exposure. It lacks information on individual exposure, which may be affected by personal measures/lifestyle.

**Answer**: Thank you for this comment. This data is based on textual medical records of patients in the Assuta eye Clinic. The treating ophthalmologist does not collect lifestyle records. This is a limitation of a retrospective study - this limitation was added to the discussion paragraph lines 130-135 of the manuscript.

Comment 4
Several studies show the correlation of different air pollutants with AMD prevalence. Since this study aimed to better understand the interactions between air pollution and the development of AMD, it would be more relevant to study the progression of AMD with variations in air pollution (and other factors) over a longer period of time.

**Answer** - This study is based on data of AMD patients, which are treated on average for **over 10 years in Maccabi health care**. AMD is believed to develop over a very long period. We cannot however always see the progression of the disease, also since it’s a degenerative disease, which of its nature becomes worst with time. While we could theoretically compare treated patients to others that were not treated to better understand the development of AMD over time, we don`t have data on untreated patients. Thus, although the study proposed by the reviewer is very important, we cannot apply it in practice, since all patients in the data are patients that got treated and also get older (worst) with time. For such an analysis, we would need to conduct a comparative study, and possibly compare (poorer) countries where AMD is treated to those where it is not. This analysis is over the scope of the current study. Also, in many poor countries, the population is more rural, and in some cases the air pollution is lower. Other research on this topic have found that regardless of treatment, visual acuity (as an outcome of treatment) decreases with time – probably as a result of photoreceptor loss^[2]^.

**Comment**

The discussion should include a comprehensive compilation of similar studies in other countries.

**Answer**: Thank you for this comment. Following the reviewer`s comments we added to the introduction a broader discussion on the prevalence of AMD and its relation to air pollution in different countries and studies. These are found in lines 77-85 of the manuscript.

MINOR COMMENTS
Comment1
Lines 192-193:
“It also surpassed the two standard deviations limits for PM10 and in the negative direction for O3”.
The data shows that PM10 surpassed the two standard deviations limits in both directions, similar to O3. Please correct the above statement with appropriate explanation.

Answer- that you for this comment, the text was corrected.

Comment 2
Please include shuffled correlation data for all pollutants.

**Answer – Thank you for this important comment**. We added to the SI this analysis which show a clear effect of air pollution. One must note that we first normalized all the different air pollution types to a [0-1] scale then averaged them. The effect as added to the SI section and the corresponding code were also uploaded to the project repository.

Comment 3
Lines 224-226:
“To conclude, in these separate analyses, the coefficients of the air pollution gases in
the univariate regression were all positively associated with an increase in the rate of
neovascular age-related macular degeneration”.

**Answer** - The above statement should be rephrased to “increase in the number of neovascular age-related macular degeneration”.

6. PLOS authors have the option to publish the peer review history of their article ([what does this mean?](https://journals.plos.org/plosone/s/editorial-and-peer-review-process#loc-peer-review-history)). If published, this will include your full peer review and any attached files.


If you choose “no”, your identity will remain anonymous, but your review may still be made public.


**Do you want your identity to be public for this peer review?** For information about this choice, including consent withdrawal, please see our [Privacy Policy](https://www.plos.org/privacy-policy).

Reviewer #1: No

Reviewer #2: No

[NOTE: If reviewer comments were submitted as an attachment file, they will be attached to this email and accessible via the submission site. Please log into your account, locate the manuscript record, and check for the action link "View Attachments". If this link does not appear, there are no attachment files.]

While revising your submission, please upload your figure files to the Preflight Analysis and Conversion Engine (PACE) digital diagnostic tool, <https://pacev2.apexcovantage.com/>. PACE helps ensure that figures meet PLOS requirements. To use PACE, you must first register as a user. Registration is free. Then, login and navigate to the UPLOAD tab, where you will find detailed instructions on how to use the tool. If you encounter any issues or have any questions when using PACE, please email PLOS at [figures@plos.org](mailto:figures@plos.org). Please note that Supporting Information files do not need this step.

1. <https://pubmed.ncbi.nlm.nih.gov/33116384/>
2. Neovascular age-related macular degeneration: A review of findings from the real-world Fight Retinal Blindness! Registry, Nguyen V, Barthelmes D, Gillies MC.Clin Exp Ophthalmol. 2021 Sep;49(7):652-663).
