# Supplementary material for: Exposure to air pollutants contributes to increased rate of neovascular age-related macular degeneration in Israel
Source: PLoS One. 2025 Apr 18;20(4):e0317436. doi: 10.1371/journal.pone.0317436 (PMC12007707; doi:10.1371/journal.pone.0317436)
Supplement: Attachment — Submitted filename: Response to Reviewers.docx [file pone.0317436.s011.docx]

Dec. 9, 2024

Dear Reviewers

Re: PONE-D-23-40090R1, "Exposure to air pollutants contributes to increased rate of neovascular age-related macular degeneration in Israel"

Thank you for the detailed feedback.

We appreciate the time and effort taken by the reviewers to evaluate it.

We are happy that both reviewer 2 and reviewer 3 have stated that "***All comments have been addressed***", and also that "***The authors have responded to all the comments to the best of their ability and made corrections wherever needed***" (reviewer 2) and also said that "***This is a very important topic. Faced with the impact of air pollution on health, this article has done a very complete research explanation.***"

Reviewer 3 raised some points that need to be explained and improved, which are answered and corrected below as a point-by-point response letter to the referees’ comments, describing the changes that were made in the revised manuscript (marked red).

Yours sincerely,

Alon Sela (on behalf of all authors of PONE-D-23-40090R1)

## Reviewer's Responses to Questions

**Comments to the Author**

If the authors have adequately addressed your comments raised in a previous round of review and you feel that this manuscript is now acceptable for publication, you may indicate that here to bypass the “Comments to the Author” section, enter your conflict of interest statement in the “Confidential to Editor” section, and submit your "Accept" recommendation.

Reviewer #2: **All comments have been addressed**

Reviewer #3: **All comments have been addressed**

Have the authors made all data underlying the findings in their manuscript fully available?

Reviewer #2: **Yes**

Reviewer #3: **Yes**

Is the manuscript presented in an intelligible fashion and written in standard English?

Reviewer #2: **Yes**

Reviewer #3: **Yes**

**Additional points raised by reviewer 3 and their corrective action.**

**Introduction**

1. There have been many studies on this topic in the past, and the author should clarify what is innovative or different from previous studies.

**Thank you for this important comment.** While we compared previously to some degrees the other studies, indeed we see this comparison was not deep enough. We added following the comment the following text to the discussion.

As to these previous studies, the link between air pollution and AMD in [17], examined PM2.5 exposure using satellite-based remote sensing but ignored a known bias from sensing air pollution in upper atmospheric layers. In contrast [18], focused on ground-level traffic-related pollutants (NO_2_ and CO), aligning with our approach, but excluded a broad set of pollutants such as O_3_, No_x_, SO_2,_ PM_10_ and PM_2.5_. Study [19] investigated natural dust storms, emphasizing non-anthropogenic factors. The large-scale Chinese study [20], focused solely on PM_2.5_, while the Korean study [21] included O_3_, NO_2_, CO, and PM10 but excluded No_x_, SO_2_ and PM_2.5_. Notably, as in our case, the Korean study suggested protective effects of O_3_ by prevention of UV exposure, but did not elaborate on UV-induced DNA damage pathways. Differences in climate and genetic diversity between Korea and Israel further distinguish these studies.

The Canadian study [22] identified PM_2.5_ as the sole AMD-related pollutant, potentially due to its linear analysis method and reliance on self-reported AMD stages and ignored the other air pollutants. By comparison, the UK study [23] used (as we did) ophthalmologist-diagnosed AMD based on OCT imaging (and not self-reported questionnaires), finding PM_2.5_ linked to higher self-reported AMD rates, while No_x_, NO_2_ and PM_10_ were associated with retinal anomalies typically linked to AMD, though requiring expert analysis for confirmation.

Our study stands and contributes to the growing evidence on air pollution's detrimental effects on AMD. It analyses the full and comprehensive set of pollutant, while defining AMD based on OCT imaging rather than self-reports. Unlike remote sensing methods, the ground-level pollution measurements offer greater accuracy.

As air pollution's impacts extend beyond the known global warming effects and as we show here (and by others) include real and concrete vision impairments for older adults. With the expected growth in life expectance and the growing levels of air pollution, further attention to this issue is warranted.

**Material and Methods**

2. Is the air pollution data collected only from 2016 to 2019 sufficient to represent the air pollution exposure of the subjects in this study? Has sensitivity analysis been performed?

**Thank you for this comment**! AMD and air pollution were indeed collected between 2015-2020 (AMD) / 2016-2019 (Air pollution). Following the reviewer`s comment, we added the following paragraph to the discussion section.

One needs to notice that AMD and air pollution were collected between 2015-2020 (AMD) / 2016-2019 (Air pollution). This period does not permit a true longitude study of air pollution over many rears. Nevertheless, since air pollution is also highly correlated with the type of the location (large city blocks, small villages in rural regions or industrial zones), changes in which rural regions become highly industrial require tens of years.

Future studies should inspect longer periods, and possibly, check for the AMD rates in regions which were once rural, and later became highly industrials. It might be challenging however to find such rejoins since industrial rejoins tend to grow at the peripheral sides of cities and less in fully rural places.

3. Are there corrections for personal health or disease factors? For example, diabetes, high blood pressure, frequency of eye use in work or life, UV exposure, etc.?

**Thank you for raising this point.** Since the main risk factors for the development of neovascular AMD are age, genetic background such as CFH and smoking. Diabetes is not a risk factor and HTN has a marginal effect, “eye use” have no known effect. We corrected for age which is the most significant factor.

**Results**

Please improve the quality of figures.

We have improved the quality of images and all images went through the Preflight Analysis and Conversion Engine (PACE) digital diagnostic tool, [https://pacev2.apexcovantage.com/](https://track.editorialmanager.com/CL0/https:%2F%2Fpacev2.apexcovantage.com%2F/1/010f01932c0b8a3b-f3d4439e-c62e-46ad-97fb-994567a62b9f-000000/_iLXrdVWTr72O6PpOBeF8JIf4bAeBkpIV_oA9PPNQhw=185).

**Discussion**

5. The same question, whether there are the same or different findings from past studies. What is innovativeness.

**Thank you for this comment** – please see comment 1 above where we include also an answer to this comment. The discussion includes a new comparison between main articles on the topic and our work, and shows the additional importance of our findings, not only by looking at another population, but mostly through the use of a larger set of air pollution particles, defining AMD band OCT + ophthalmologist inspection and the use of land-based tools (and not remote sensing tools) to estimate ground level air pollution.

6. In addition to exploring the issues of air pollution and health, this study should stand on a higher vantage point and make possible suggestions for policy or health countermeasures.

We totally agree on this point. THANK YOU!

This   is a major issue and this is one of the reasons we chose to perform this study.  We added to the manuscript the following paragraph in the discussion section addressing this important issue hoping to raise public and decision maker’s awareness.

A growing body of literature is accumulating from different countries, ethnicities and climates, pointing to the harmful effect of air pollution and its clear influence on AMD. While reducing air pollution is a major concern to global warming, increased rates of blindness at old age needs to alert decision makers no less, especially with prolonged lifespan. Further investment in research related to this alerting topic needs to be concluded, to better understand the exact biological paths by which air pollution damages the retina. This may also shed light on the pathogenesis of this complex degenerative disease and possibly to find additional treatments. The role of countries and health organization is to raise awareness to this additional effect of air pollution and help to further reduce the rates of this blinding disease and by this - to reduce long-term treatment expenses.

We have uploaded our figure files to the Preflight Analysis and Conversion Engine (PACE) digital diagnostic tool, [https://pacev2.apexcovantage.com/](https://track.editorialmanager.com/CL0/https:%2F%2Fpacev2.apexcovantage.com%2F/1/010f01932c0b8a3b-f3d4439e-c62e-46ad-97fb-994567a62b9f-000000/_iLXrdVWTr72O6PpOBeF8JIf4bAeBkpIV_oA9PPNQhw=185).
